# Supplementary material for: Dissecting Rubella Placental Infection in an In Vitro Trophoblast Model
Source: Int J Mol Sci. 2023 Apr 26;24(9):7894. doi: 10.3390/ijms24097894 (PMC10178045; doi:10.3390/ijms24097894)
Supplement: Supplementary file 1 [file ijms-24-07894-s001.zip › ijms-2306865-supplementary.pdf]

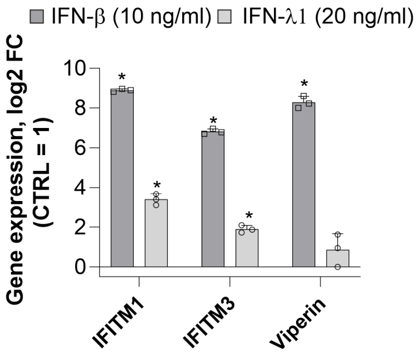

Supplement Figure S1. Quantification of mRNA expression of indicated ISGs by qPCR after incubation with indicated IFNs for 24 hours. Expression data were normalized to β-actin mRNA in the comparative cycle threshold method (ΔΔCt). All data (n=3) are shown as mean + SD, * $p < 0.05$ as determined by unpaired Student's t test.

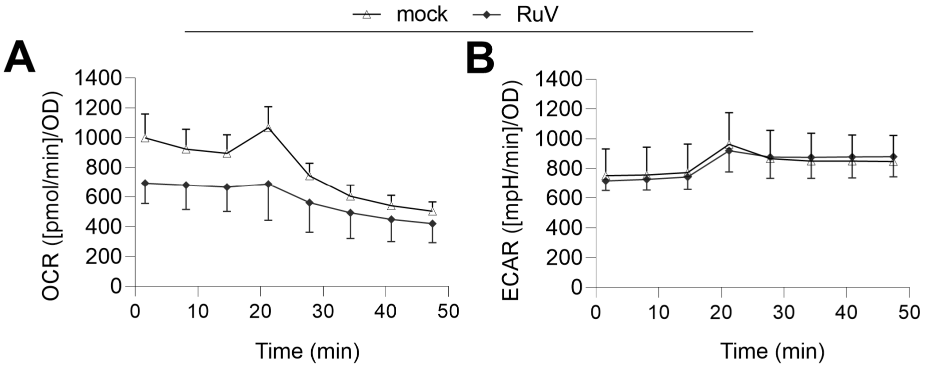

Supplement Figure S2. Measurement file of (A) OCR and (B) ECAR. The cell energy phenotype kit was used for mock- and RuV-infected BeWo at 72 hpi.
